# ERG Deregulation Induces PIM1 Over-Expression and Aneuploidy in Prostate Epithelial Cells

**DOI:** 10.1371/journal.pone.0028162

**Published:** 2011-11-30

**Authors:** Vera Magistroni, Luca Mologni, Stefano Sanselicio, James Frances Reid, Sara Redaelli, Rocco Piazza, Michela Viltadi, Giorgio Bovo, Guido Strada, Marco Grasso, Manuela Gariboldi, Carlo Gambacorti-Passerini

**Affiliations:** 1 Department of Clinical Medicine, University of Milano-Bicocca, Monza, Italy; 2 Department of Experimental Oncology and Molecular Medicine, Fondazione IRCCS Istituto Nazionale dei Tumori, Milano, Italy; 3 Unit of Molecular Genetics of Cancer, IFOM Foundation, Milano, Italy; 4 Department of Pathology, S. Gerardo Hospital, Monza, Italy; 5 Urology Division, Bassini ICP Hospital, Milano, Italy; 6 Department of Urology, San Gerardo Hospital, Monza, Italy; 7 Section of Haematology, San Gerardo Hospital, Monza, Italy; University of Saarland Medical School, Germany

## Abstract

The ERG gene belongs to the ETS family of transcription factors and has been found to be involved in atypical chromosomal rearrangements in several cancers. To gain insight into the oncogenic activity of ERG, we compared the gene expression profile of NIH-3T3 cells stably expressing the coding regions of the three main ERG oncogenic fusions: TMPRSS2/ERG (tERG), EWS/ERG and FUS/ERG. We found that all three ERG fusions significantly up-regulate PIM1 expression in the NIH-3T3 cell line. PIM1 is a serine/threonine kinase frequently over-expressed in cancers of haematological and epithelial origin. We show here that tERG expression induces PIM1 in the non-malignant prostate cell line RWPE-1, strengthening the relation between tERG and PIM1 up-regulation in the initial stages of prostate carcinogenesis. Silencing of tERG reversed PIM1 induction. A significant association between ERG and PIM1 expression in clinical prostate carcinoma specimens was found, suggesting that such a mechanism may be relevant *in vivo*. Chromatin Immunoprecipitation experiments showed that tERG directly binds to PIM1 promoter in the RWPE-1 prostate cell line, suggesting that tERG could be a direct regulator of PIM1 expression. The up-regulation of PIM1 induced by tERG over-expression significantly modified Cyclin B1 levels and increased the percentage of aneuploid cells in the RWPE-1 cell line after taxane-based treatment. Here we provide the first evidence for an ERG-mediated PIM1 up-regulation in prostate cells *in vitro* and *in vivo*, suggesting a direct effect of ERG transcriptional activity in the alteration of genetic stability.

## Introduction

Distinct alterations of the transcription factor ERG, an ETS-related gene, are observed in a variety of cancers. Aberrant expression of full-length ERG protein has been found in acute myeloid leukaemia and acute T-lymphoblastic leukaemia [1], [2]. Chromosomal rearrangements driving the formation of EWS/ERG and FUS/ERG fusion proteins have been described in a subset of Ewing sarcoma [3], [4] and in acute myeloid leukaemia [5]–[7]. A significant part of ERG sequence is lost in these fusions [4], [7], as the entire NH_2_-terminal pointed-domain is missing. The EWS and FUS genes encode for two related RNA-binding proteins that lose their RNA binding domains during translocation with ERG. The resulting fusion proteins maintain the EWS or the FUS transactivator domains (able to bind to RNA polymerase II) and both acquire the ERG-derived DNA-binding domain [8]–[10]. In 2005 the most frequent genetic alteration involving ERG was found in prostate cancer (PCa), where the 5′-untrascribed region of the prostate-specific and androgen-responsive TMPRSS2 gene is fused to ERG in approximately 50% of PCa cases [11]. As a consequence of this genetic alteration the TMPRSS2 promoter region determines the inappropriate and androgen-driven expression of ERG in prostate cells. Several tERG alternative fusions have been identified [12]. In the most common rearrangement (T1/E4), a truncated ERG protein (tERG) that maintains its ERG DNA-binding domain is expressed. Since the first three exons are lost during rearrangement, an alternative translation initiation site from an internal ATG codon is used to translate the fusion transcript [13].

tERG, EWS/ERG and FUS/ERG fusions are all considered early events in cancer progression. However, in contrast to the established oncogenic properties of EWS/ERG and FUS/ERG [14]–[16], tERG is clearly not sufficient to generate a fully transformed phenotype *in vitro* and *in vivo* [17]–[20], although it seems able to induce pre-neoplastic changes. Despite this, when tERG is combined with additional genetic alterations that are typical of PCa, such as aberrant PI3K signalling activation or enhanced androgen receptor signalling, it can lead to an aggressive disease [17], [20]. All these lesions are not able to induce tumoral transformation in prostate cells as single events, but the synergism between them and the tERG rearrangement is sufficient to promote PCa progression [17]. It is thus reasonable to hypothesize that since tERG is an early event found at high frequency in PCa specimens but lacks strong oncogenic features, it may promote the progression towards a neoplastic phenotype by favouring the achievement of secondary alterations. In line with this, it has recently been shown that tERG over-expression can induce an increase in the amount of double strand breaks in prostate cells, thus creating a putative “error-prone” phenotype [21]. However, this effect is not linked to a modified expression of DNA repair genes, while it seems associated with the direct binding of tERG to Poly(ADP-Ribose)Polymerase 1 (PARP-1) [21].

In this study we show that expression of the coding regions of the three main ERG rearrangements (EWS/ERG, FUS/ERG and tERG) up-regulate PIM1, an oncogene found increased in a broad range of tumours of both epithelial and haematological origin, including PCa [22]–[27] where its over-expression has been associated with genomic instability [28]–[30]. To investigate the link between tERG and PIM1 in prostate cancer, we modulated ERG expression in the non malignant RWPE-1 prostate cell line. Our results reveal that PIM1 is a direct target of tERG and that this effect can favour genomic instability in a pre-cancerous environment.

## Results

### Overexpression of ERG oncogenic forms in NIH-3T3 cells

**Figure 1:**
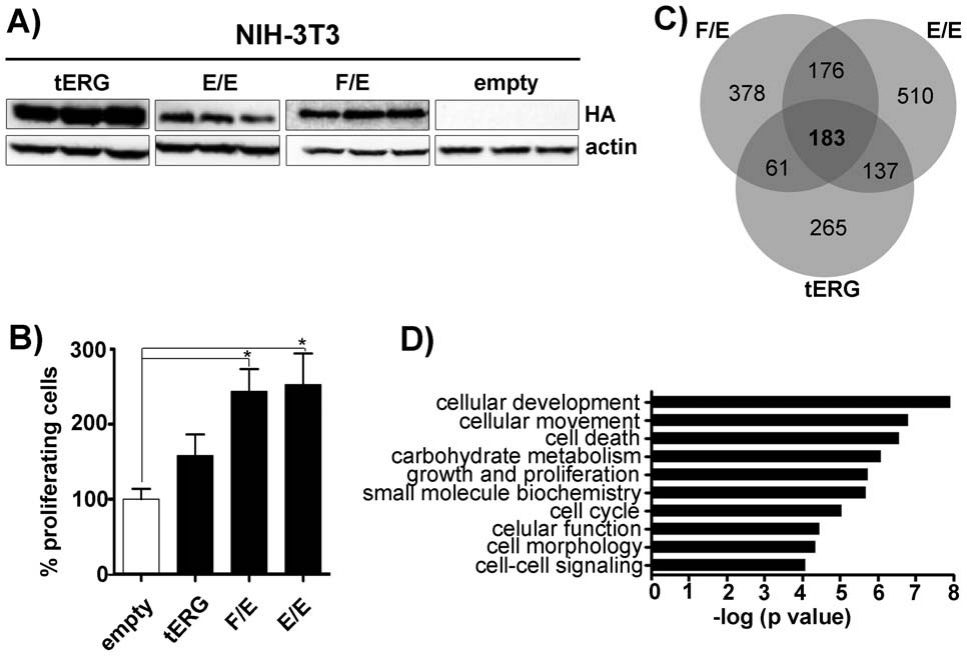
Overexpression of HA-tagged TMPRSS2/ERG (tERG), EWS/ERG (E/E) and FUS/ERG (F/E) in NIH-3T3 cells. **a**) Anti-HA immunoblot of NIH-3T3 whole cell lysates. Each transfection was performed in triplicate. Actin was used as a loading control. **b**) Evaluation of NIH-3T3 proliferative activity through [3H]Thymidine incorporation assay after 48 hrs of growth. Data for each ERG form represent the average of the results of the three independent populations and are normalized against the average of NIH-3T3_empty vector cells (*p<0.05). **c**) Venn diagrams of the transcription profiles of NIH-3T3 transfectants considering genes with a cut-off ≥1.5 fold differential expression compared to control cells (NIH3T3-empty). **d**) Graphic representation of the top 10 highly significant molecular and cellular function categories identified by the common 183 deregulated genes. Categories ranking was obtained by Ingenuity Pathway Software. Significance refers to the −log(p-value), which was obtained by the Ingenuity program using a right-tailed Fisher's exact test.

The coding regions of human tERG (T1/E4) [12], FUS/ERG (isoform B) [9] and EWS/ERG (isoform 1e) [10] were inserted into the phCMV2 vector. The derived phCMV2_HA_tERG, phCMV2_HA_FUS/ERG and phCMV2_HA_EWS/ERG plasmids were introduced into the highly transfectable NIH-3T3 fibroblast cell line. In order to obtain reliable results, three independent transfections were performed for each plasmid. Western blot analysis of stable transfectants (Fig. 1A) showed the presence of the ectopic proteins in all the populations. The over-expression of ERG in all the tERG-positive populations was confirmed also by quantitative Real-Time PCR (13459±690.4 fold change compared to empty vector-transfected cells; data not shown). Tritiated-thymidine incorporation assay revealed that tERG over-expression is not sufficient to induce a significant increase in the proliferative potential of NIH-3T3 cells compared to control cells transfected with empty vector (hereafter referred to as empty). Conversely, we observed a significant hyper-proliferative activity for the EWS/ERG and the FUS/ERG fusions (p<0.05) (Fig. 1B).

### Gene expression signature in NIH-3T3 cells transfectants

To dissect the effect of the three ERG fusions in a homogeneous cellular model, we analyzed the gene expression signature for each NIH-3T3 transfectant using a microarray-based gene expression profiling approach covering more than 28,000 genes (see Materials and Methods for detail). The results showed that, compared with control cells, tERG, FUS/ERG and EWS/ERG induced a ≥1.5-fold significant deregulation of 646, 798 and 1006 genes, respectively. Some of the deregulated genes were randomly chosen, and the expression was measured by quantitative Real-Time-PCR. The results were compatible with microarray data (Table 1). Venn diagrams reveal the presence of 183 common deregulated genes (Fig. 1C). Among them we observed up-regulation of MMP3 and CDH5, previously described as ERG target genes [18], [31], thus confirming the reliability of our model (Table 1). We used the Ingenuity Pathway Analysis (IPA) to annotate the functional categories of the significantly deregulated genes. In Figure 1D the first 10 top-ranking molecular and cellular function categories identified by the 183 commonly deregulated genes are shown.

**Table 1:** Validation of gene expression deregulation through quantitative Real-Time PCR in NIH-3T3 transfectants.

GENE (official gene symbol)	empty	tERG	EWS/ERG	FUS/ERG
Cdh5	1	(↑) +33.7	(↑) +8.1	(↑) +12.4
Mmp3	1	(↑) +63.8	(↑) +79.86	(↑) +243.9
Edn1	1	(↑) +31.9	(↑) +14.7	(↑) +21.5
Irs1	1	(n) +1.2	(↑) +2.07	(↑) +2.49
Egln3	1	(↑) +4.6	(↑) +4.3	(↑) +2.3
Dpysl3	1	(↑) +5.2	(↑) +7.2	(↑) +7
Cyp1b1	1	(n) +1.1	(↑) +9.7	(↑) +5
Has2	1	(↓) −1.9	(↓) −6.9	(↓) −7.3
Ednra	1	(↑) +4.8	(↑) +11.4	(↑) +42
Ccl9	1	(↑) +9	(↑) +3.1	(↑) +2.1
Gpr149	1	(↑) +5.7	(↑) +7	(↑) +5.7
Id1	1	(↓) −9.2	(n) −1.9	(n) −1.3

Significant up- (↑) or down- (↓) regulation obtained by microarray analysis is shown alongside quantitative Real-Time PCR fold change values for a direct comparison. n = no change.

### Co-expression of ERG and PIM1 in prostate cancer

**Figure 2:**
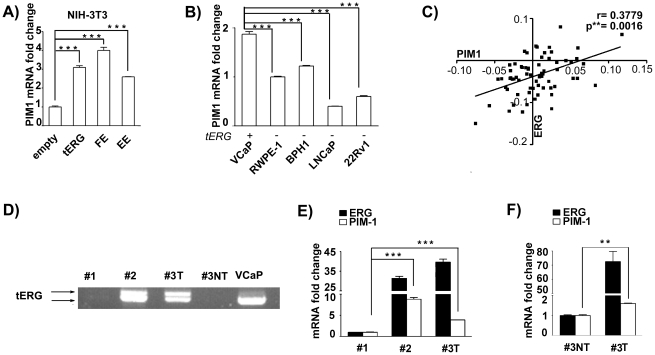
Significant co-expression of ERG and PIM1. **a**) PIM1 expression fold change in NIH-3T3 transfectants compared to empty-vector cells, as determined by Quantitative Real-Time PCR (*** = p<0.001). **b**) Quantitative Real-Time PCR for PIM1 in human prostate cell lines. PIM1 expression, relative to the non-malignant RWPE-1 cell line is visualized in the graph (*** = p<0.001). tERG positivity is shown. **c**) Correlation between ERG and PIM1 expression in prostate samples from the Kunderfranco et al dataset [34]. The Spearman rank correlation test was used to estimate the correlation significance (p = 0.0016; r = 0.3779). **d**) cDNA prepared from human prostate samples (tumors #1 and #2, plus a matched tumoral/non-tumoral pair [#3T/#3NT]) and from the tERG-positive VCaP cell line, was amplified as described in materials and methods. The arrows indicate the tERG amplified products. **d–e**) Quantitative Real-Time PCR for ERG and PIM1 in prostate cancer samples. Relative ERG and PIM1 expression compared to the tERG-negative prostate cancer #1 (**d**) or to the non-tumoral sample #3NT (**e**) is reported. ** = p<0.01; *** = p<0.001; T = tumoral; NT = non-tumoral.

Among the genes significantly affected by all three ERG fusions (Fig. 1C), we noted the up-regulation of PIM1, an oncogene whose expression is increased in tumors from both haematological and epithelial origin, including prostate cancer [22], [32], [33]. These data were further confirmed by quantitative RT-PCR on NIH-3T3 transfectants for all the ERG oncogenic forms (Fig. 2A). In addition, examination of prostate cell lines showed a highly significant difference in PIM1 expression between the tERG positive and negative cell lines (p<0.001) (Fig. 2B). We then found a significant (p<0.01) correlation between ERG expression and PIM1 up-regulation in a prostate cancer dataset [34], further confirming the link between ERG and PIM1 in prostate cancer (Fig. 2C). The presence of the tERG fusion in three distinct prostate cancer samples and in a matched non-tumoral adjacent tissue from patient #3 was then evaluated (Fig. 2D). Samples #2 and #3 proved to be tERG positive, with the corresponding non-tumoral sample (#3NT) being negative. Sample #2 and #3 (tERG positive) showed significantly higher PIM1 expression than sample #1 (Fig. 2E). In addition, tumour tissue from patient #3 displayed higher PIM1 and ERG levels than the corresponding autologous non-malignant prostate (Fig. 2F).

### tERG directly binds to PIM1 promoter in RWPE-1 cells and modulates PIM1 expression

**Figure 3:**
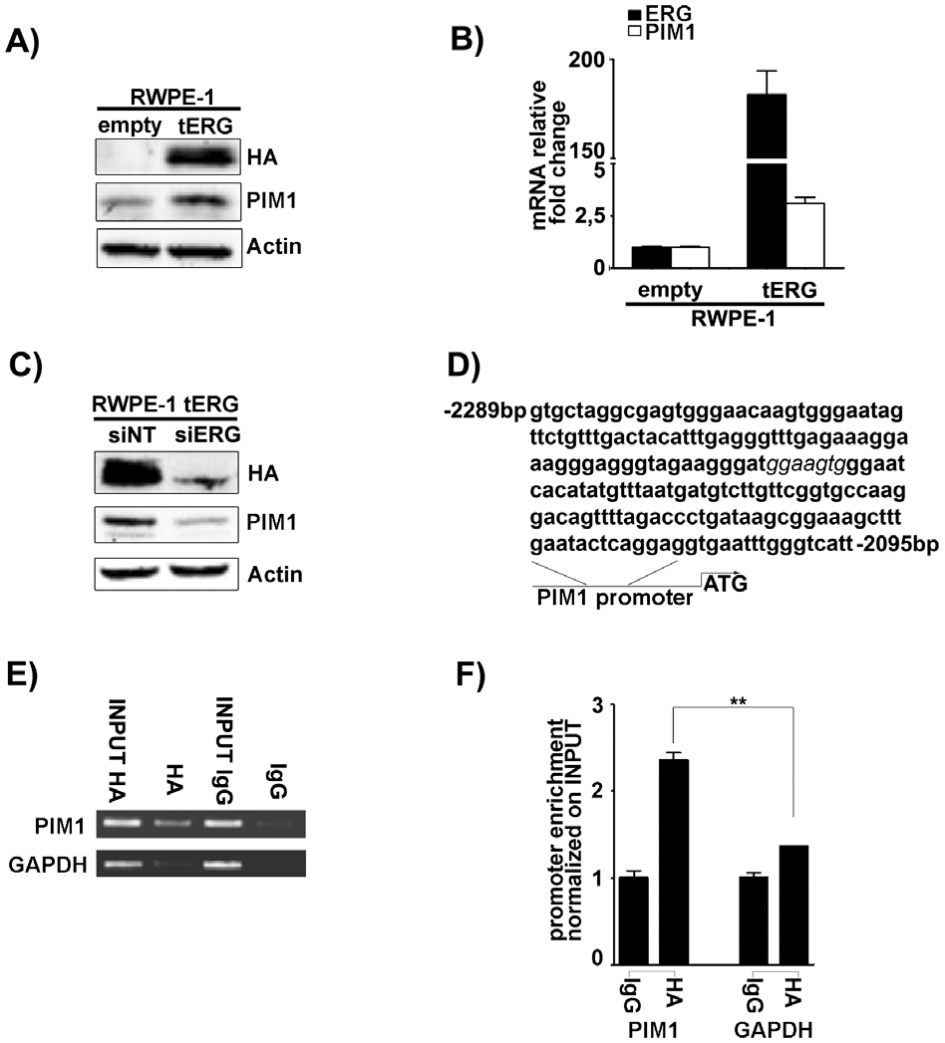
ERG dependent PIM1 induction in RWPE-1 cells. **a–b**) PIM1 over-expression in RWPE-1 transfected with HA-tagged TMPRSS2/ERG coding region (RWPE-1_tERG) as evidenced by immunoblot (**a**) and Real-Time quantitative PCR (**b**). Actin is shown as a loading control. ERG and PIM1 fold changes were normalized against empty-vector cells. **c**) siRNA knockdown of ERG (siERG) in RWPE-1_tERG induces a decrease in PIM1 levels compared to non-targeting siRNA (siNT). **d**) Sequence of the 194 bp-long PIM1 promoter region amplified in ChIP analysis. The 7 bp putative ERG binding site identified by Transcription Element Search System (TESS) is shown in italic. The distances from the ATG starting site are presented. **e–f**) Chromatin Immunoprecipitation (ChIP) showed a significant enrichment of HA-tERG binding to PIM1 promoter compared to IgG control in RWPE-1_tERG. Glyceraldehyde-3-phosphate dehydrogenase (GAPDH) promoter was used as a negative control. ** = p<0.01.

To gain further insights into the role of tERG-mediated PIM1 induction in prostate cancer progression, we stably over-expressed tERG in the non-tumorigenic prostate epithelial cell line RWPE-1. tERG over-expression induced PIM1 both at the mRNA (3.1±0.5 fold change compared to empty cells) and protein levels (Fig. 3A–B). Furthermore, ERG silencing in RWPE-1_tERG transfectants led to PIM1 down-regulation, confirming the role of the ERG transcription factor in PIM1 induction (Fig. 3C). To address the interaction between tERG and PIM1 promoter, anti-HA chromatin immunoprecipitates (ChIP) from RWPE-1_tERG cells were subjected to quantitative RT-PCR using primers specific for the region from −*2095* bp to −*2289* bp upstream of the ATG start codon of PIM1 (NC_000006) (Fig. 3D). This 194 bp stretch includes a putative ERG binding site (GGAAGTG) as defined by the Transcription Element Search System (TESS-http://www.cbil.upenn.edu/cgi-bin/tess). ChIP analysis showed a significant enrichment of PIM1 promoter compared to a region in the GAPDH promoter where no ERG binding sites are present (Fig. 3E–F).

### RWPE-1 cells over-expressing the tERG/PIM1 axis display a slower rate of recovery after treatment with chemotherapeutic drugs

**Figure 4:**
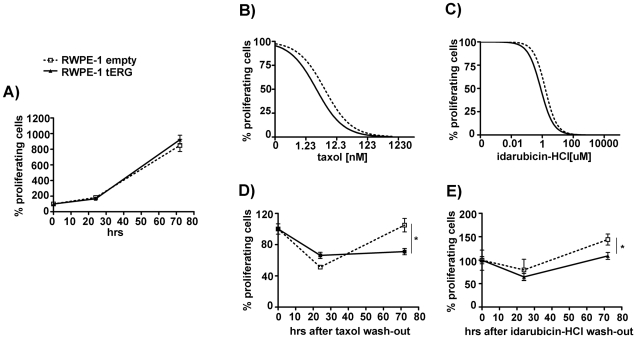
Sensitivity of RWPE-1_tERG cells to chemotherapeutic drugs. **a**) Proliferative potential of tERG over-expressing and control (empty) cells measured by thymidine uptake assay up to 72 hrs growth in standard conditions. **b–c**) 72 hours treatment of RWPE-1 transfectants with taxol or idarubicin-hydrochloride. Proliferative potential was measured by thymidine uptake assay. **d–e**) Re-growth of RWPE-1 transfectants after 10 nM taxol (**d**) or 0.5 µM idarubcicin-hydrochloride (**e**): following 24-hrs treatment, drugs were removed and the cells allowed to grow for additional 72 hours. Proliferation of cells collected at the time of wash-out is set as 100%. * = p<0.05.

tERG over-expression did not confer a growth advantage to RWPE-1 cells in standard conditions (Fig. 4A). PIM1 is an oncogene whose over-expression has been associated to the acquisition of drug resistance in prostate cancer [35]–[37]. As evaluated by IC50 values (Fig. 4B–C), RWPE-1_tERG transfectants did not display increased resistance to taxane, nor to anthracycline-based drugs (IC50 empty vs tERG: Idarubicin-HCl 1.57 uM vs 0.79 uM; Taxol 5.05 nM vs 2.71 nM). However the quantification of re-growth of transiently treated cultures showed an early protection in taxane-treated cells, followed by a later decrease in the proliferative rate for tERG positive cells compared to control (p<0.01) (Fig. 4D–E). The tERG/PIM1 axis is thus linked to a lower ability to recover from DNA-damaging drugs, and in particular from drugs blocking the G2/M switch, in the non malignant RWPE-1 cell line.

### ERG-dependent PIM1 induction deregulates cyclin B1 level and favours the appearance of aneuploidy

**Figure 5:**
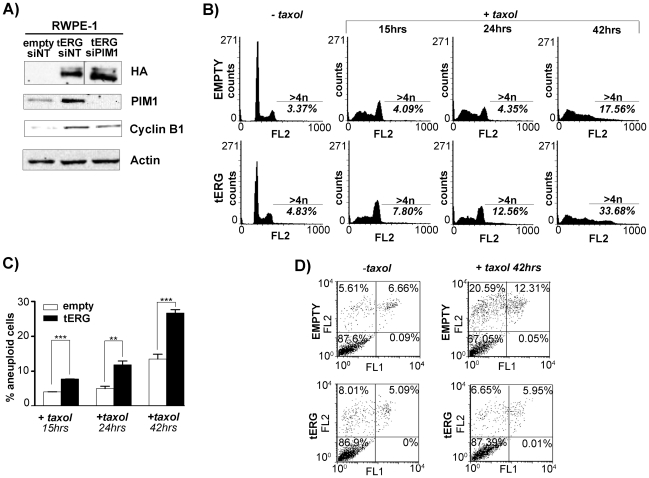
The tERG/PIM1 axis favours aneuploidy. **a**) Immunoblot assay demonstrates Cyclin B1 increase in RWPE-1_tERG cells. siRNA knockdown of PIM1 (siPIM1) reduces this effect compared to non-targeting siRNA (siNT). The same amount of whole cell lysates were loaded in two different polyacrilamide gels. The tERG_siPIM1 sample for the HA immunoblot was loaded in a different position compared to the gel for PIM1, Actin and Cyclin B1 blot **b**) Flow cytometric analysis of non-synchronized propidium iodide stained cells. Treatment with taxol for 15/24/42 hours induces an important increase in the >4n fraction of RWPE-1_tERG compared to empty cells. **c**) Percentage of >4n cells after taxol treatment obtained from the average of at least two independent experiments. ** = p<0.01, *** = p<0.001. **d**) Annexin-V assay was carried out to evaluate apoptosis after 42 hrs of 10 nM taxol treatment for RWPE-1 transfectants. The percentage of cells in each quadrant is shown.

The slower recovery of RWPE-1_tERG cells after treatment with chemotherapeutic drugs, prompted us to investigate whether the tERG/PIM1 axis could affect genetic stability in early phases of prostate cancer progression. It has been previously described that PIM1 over-expression can induce aneuploidy in RWPE-1 cells and increases cyclin B1 levels thus deregulating the G2/M phase of the cell cycle [29]. We first assessed cyclin B1 protein amount in RWPE-1 transfectants. We found that tERG-positive cells display higher cyclin B1 levels compared to empty cells, and that PIM1 silencing reduces its over-expression (Fig. 5A). Flow-cytometry (FACS) analysis of propidium iodide-stained RWPE-1 cells did not show differences in cell cycle profile between empty and tERG transfectants (Fig. 5B). However, we observed a significant increase in the amount of >4n cells after taxol-induced microtubule stress in tERG-positive compared to empty cells (Fig. 5B–C). Taxol treatment of RWPE-1 transfectants revealed an increased viability of tERG cells compared to empty cells after 42 hours of treatment (Fig. 5D). Furthermore, PIM1 silencing in RWPE-1_tERG cells caused a moderate decrease in the aneuploid portion of RWPE-1_tERG taxol-treated cells (from 7.03±3.2% in the non targeting silencing to 4.84±0.64% in the PIM1 specific silencing after 24 hours treatment; data not shown) These data suggest a direct effect of the tERG/PIM1 axis in the alteration of genomic stability in pre-malignant prostate cells.

## Discussion

The ETS family of transcription factors, comprising more than 20 genes, has been associated to the pathogenesis of several cancers, often due to gene rearrangements involving the ETS domain. ERG is one of the most affected members of the family, being involved in several chromosomal translocations in different tumors. Here we used a homogeneous cellular model to compare the effect of the three main ERG oncogenic forms found in tumoral specimens and derived from non canonical chromosomal rearrangements: tERG, EWS/ERG and FUS/ERG. Gene expression analysis of NIH-3T3 transfectants revealed a minimum overlap in the affected genes despite the maintenance of the ERG DNA binding domain in all the three fusions. The 183 commonly deregulated genes represented 28.3%, 22.9% and 18.1% of the entire differentially expressed gene set for tERG, FUS/ERG and EWS/ERG, respectively.

This study shows for the first time that tERG, EWS/ERG and FUS/ERG can significantly up-regulate the transcription of the serine/threonine kinase PIM1 in the NIH-3T3 cell line. PIM1 was of immediate interest because it is supposed to play an oncogenic role in several types of tumours, including prostate cancer [22], [32], [33]. Since we found a significant correlation between ERG-expression and PIM1 upregulation in a previous published prostate cancer data-set [34], we evaluated several prostate cancer specimens and prostate cancer cell lines for ERG and PIM1 expression, confirming a significant correlation between ERG and PIM1 mRNA levels. Quantitative Real-Time PCR analysis of an ERG-positive tumour and the corresponding non-tumoral adjacent tissue validated the connection between ERG and PIM1 at the transcriptional level. The association between ERG and PIM1 expression in clinical specimens suggests that such a mechanism may be relevant in ERG induced prostate tumorigenesis *in vivo*.

Since tERG is considered an early event in PCa progression [38], [39], we modulated tERG expression in the non-tumorigenic immortalized RWPE-1 prostate cell line. We showed here that PIM1 levels changed in accordance to tERG amount also in a non-malignant prostate model and that tERG can directly bind to PIM1 promoter. Since it has previously been observed that PIM1 over-expression can exert a pro-survival activity in prostate cancer cells treated with chemoterapeutic drugs [40], [41], we tried to assess whether this phenomenon can be observed also in the non-tumoral RWPE-1 model. Our results showed that the tERG/PIM1 axis is unable to induce sustained resistance to taxane or anthracycline-based drugs in pre-malignant lesions, but significantly increases the sensitivity of non-tumoral cells in a re-growth assay after drug treatments. Since sensitivity to chemotherapeutic drugs can be a consequence of increased genetic instability in cells, we tried to understand whether the tERG mediated PIM1 induction could cause some form of genomic instability. PIM1 is known to be important for the maintenance of a stable mitotic apparatus and for the proper dynamics of spindles [28], [42]. We therefore looked at the development of aneuploidy following treatment with a cytotoxic drug. Cell cycle analysis of our RWPE-1 transfectants did not show significant differences between tERG and empty cells. Despite this, treatment with paclitaxel, a taxane-based microtubule-interacting drug, induces a significant increase in the >4n population in the tERG positive cells, suggesting an involvement of the tERG/PIM1 axis in the alteration of genomic stability after cellular stresses. The central role of PIM1 in this phenomenon was then confirmed through PIM1 transient silencing in the paclitaxel-treated tERG cells. In addition we demonstrated that tERG up-regulation can influence Cyclin B1 levels, an event previously associated with the appearance of aneuploidy in PIM1 over-expressing prostate cells [29]. We here showed that cyclin B1 increase in RWPE-1 transfectants is mostly driven by PIM1 up-regulation, although it is not possible to exclude additional regulatory mechanisms mediated by tERG over-expression. In fact, transient PIM1 silencing was able to modulate Cyclin B1 levels only in part.

It is widely accepted that the appearance of aneuploidy in non-malignant cells can provide a favourable environment for the acquisition of additional mutations. The stress caused by aneuploidy can promote genomic changes and induces genetic instability thus playing a causative role in early tumourigenesis [43]. We found that non-tumoral tERG-positive cells are more sensitive to the acquisition of aneuploidy after taxane-based treatment thanks to PIM1 induction. Furthermore, annexin-V assay showed that at early time-points after taxol treatment tERG cells display a lower level of apoptosis compared to control cells. Therefore, the aptitude of RWPE-1_tERG cells to develop aneuploidy and to avoid apoptosis at least in the initial phases after a drug-induced stress, suggests the existence of a cellular environment that favours genetic instability induced by the tERG/PIM1 axis. We propose that TMPRSS2/ERG fusion, although unable to confer growth advantage, renders epithelial cells prone to the acquisition of new mutations, which will then be selected during malignant transformation.

PIM1 expression is known to be elevated in approximately 50% of prostate cancer cases [22]. Despite many efforts in the identification of PIM1 kinase inhibitors for the treatment of PCa, little is known about the regulation of PIM1 levels in epithelial malignant cells. Here we provide the first evidence of an ERG mediated PIM1 up-regulation in a non-tumoral prostate cell line. We also suggest that a similar ERG-mediated regulation of PIM1 expression could be present for the EWS/ERG and FUS/ERG fusions. This discovery provides novel insights into the role of TMPRSS2/ERG in prostate cancer progression and describes a new mechanism for PIM1 regulation mediated by the ERG DNA binding domain.

## Materials and Methods

### Prostate tumor specimens

Three localized prostate cancer samples and a non-neoplastic paired tissue were taken freshly from total prostatectomy specimens. This study was approved by the internal Ethical Committee of San Gerardo Hospital. Pathology-certified, fully anonimized human samples were provided according to local IRB provision (San Gerardo Hospital IRB document ASG-DA-950, april 2008). Patients provided written informed consent to donate the tissues left over after diagnostic procedures.

### Cell lines

The prostate cell lines RWPE-1, VCaP, LNCaP and 22Rv1 were obtained from the American Type Culture Collection (ATCC), while the benign prostate hyperplasia (BPH-1) and the NIH-3T3 mouse fibroblast cell lines were purchased from DSMZ.

The immortalized non malignant prostate cell line RWPE-1 was maintained in keratinocyte-serum free medium supplemented with epidermal growth factor and bovine pituitary extract. BPH-1 cells were grown in RPMI supplemented with 20% fetal bovine serum, 20 ng/ml testosterone, 5 µg/ml transferrin, 5 ng/ml sodium selenite and 5 µg/ml insulin. LNCaP and 22Rv1 cells were maintained in RPMI while VCaP and NIH3T3 cell lines were maintained in DMEM. In these latter cases both RPMI and DMEM medium were supplemented with 10% FBS.

### Generation of plasmids and transfections

The cDNA of the putative translated sequence of TMPRSS2/ERG (isoform 9) [12] was obtained from the RNA of the prostate cancer cell line VCaP and inserted into the phCMV2 vector (Genlantis, CA, USA) through XhoI/NotI restriction sites to obtain the phCMV2_HA_tERG plasmid. The FUS/ERG (isoform B) [9] and EWS/ERG (isoform 1e) [10] cDNA sequences were derived from pSG5-fl-FUS/ERG and pSG5-fl-EWS/ERG respectively, kindly provided by Prof. Liu Yang [44], [45] and inserted into phCMV2 as above, to obtain phCMV2_HA_FUS/ERG and phCMV2_HA_EWS/ERG. The primers used to amplify the selected fragments for the cloning into phCMV2-HA vector were: FUS/ERG_Fw _5′_tactcgagatggcctcaaacgattatacc_3′_ , EWS/ERG_Fw _5′_tactcgagatggcgtccacggattacag_3′_ and tERG_Fw _5′_tactcgagatgaccgcgtcctcctccagc_3′_ . The reverse primer for all the ERG forms was ERG_Rw _5′_tatgcggccgcttagtagtaagtgcccagatg_3′_ . The RWPE-1 cell line was transfected with Fugene 6 (Roche, Applied Science, Germany) according to manufacturer's instruction and selected for geneticin (Invitrogen, Carlsbad, CA, USA) resistance at 0.25 mg/ml.

### Retrotranscription and Quantitative Real-Time PCR

RNA was extracted with TRIzol Reagent (Invitrogen, Carlsbad, CA, USA) and cDNA was synthesized from 200 ng of total RNA, using ‘TaqMan Reverse Transcription Reagents’ (Applied Biosystems, Foster City, CA, USA). The RNA from transfectant cells was pre-treated with DnaseI (Invitrogen, Carlsbad, CA, USA) to avoid contamination from genomic DNA. The TMPRSS2/ERG positivity was assayed on cDNA using the Fast Start Taq DNA polymerase (Roche, Applied Science, Germany) with the primers TMPRSS2-RT-f and TMPRSS2:ERG_RT-r as described in Tomlins et al. [11]. Quantitative RT-PCR was performed using Brilliant SYBR Green QPCR Master Mix (Stratagene, La Jolla, CA) on a Stratagene-MX3005P under standard conditions. All the quantitative Real-Time PCR experiments were performed in triplicate. Immediately after amplification, PCR products were analyzed by sequencing, dissociation curve analysis, and gel electrophoresis to determine specificity of the reaction. The house-keeping genes GAPDH (homo sapiens) and Gus (mus musculus) were used as endogenous references. The sequences of the primers used are reported (hs: homo sapiens, mm: mus musculus): GAPDHFw(hs) _5′_tgcaccaccaactgcttagc_3′_ and GAPDHRw(hs) _5′_ggcatggactgtggtcatgag_3′_ [46]; ERGFw(hs) _5′_cgcagagttatcgtgccagcagat_3′_ and ERGRw(hs) _5′_ccatattctttcaccgcccactcc_3′_ [11]; PIM1Fw(hs) _5′_cgagcatgacgaagagatcat _3′_ and PIM1Rw(hs) _5′_tcgaaggttggcctatctga_3′_ [47]; Hprt1Fw(mm) _5′_tcagtcaacgggggacataaa_3′_ and Hprt1Rw(mm) _5′_ggggctgtactgcttaaccag_3′_ [48]; Cdh5Fw(mm) _5′_agacacccccaacatgctac_3′_ and Cdh5Rw(mm) _5′_gcaaactctccttggagcac_3′_ [49]; Mmp3Fw(mm) _5′_aagggtggatgctgtctttgaagc_3′_ and Mmp3Rw(mm) _5′_gccatagcacatgctgaacaaagc_3′_ [50]; Edn1Fw(mm) _5′_gcgtcgtaccgtatggactgg_3′_ and Edn1Rw(mm) _5′_atgccttgatgctattgctgatg_3′_ ; Irs1Fw(mm) _5′_taggcagcaatgagggcaactc_3′_ and Irs1Rw(mm) _5′_tgaggtcctggttgtgaattg_3′_ ; Egln3Fw(mm) _5′_ggcacctgcgaggcgaccagat_3′_ and Egln3Rw(mm) _5′_tggcgaacataacctgtcccattt_3′_ ; Dpysl3Fw(mm) _5′_cgagcagcagcagtagcagcga_3′_ and Dpysl3Rw(mm) _5′_atgcctccagggatcaccatcttc_3′_ ; Cyp1bFw(mm) _5′_gcctgccactattacggaca_3′_ and Cyp1b1Rw(mm) _5′_acaacctggtccaactcagc_3′_ [51]; Has2Fw(mm) _5′_cgagtctatgagcaggagctg_3′_ and Has2Rw(mm) _5′_gtgattccgaggaggagagaca_3′_ [52]; EdnraFw(mm) _5′_tacaagggcgagctgcatag_3′_ and EdnraRw(mm) _5′_catgagggtgtagaagattgctg_3′_ ; Ccl9Fw(mm) _5′_ggccagctgggtctgcccac_3′_ and Ccl9Rw(mm) _5′_tgcccggcctggtacacccac_3′_ ; Gpr149Fw(mm) _5′_cgttgccttcgatgggaaaaa_3′_ and Gpr149Rw(mm) _5′_gctcctttgtagcttcacactca_3′_ ; Id1Fw(mm) _5′_ttggtctgtcggagcaaagcgt_3′_ and Id1Rw(mm) _5′_cgtgagtagcagccgttcatgt_3′_ ; PIM1Fw(mm) _5′_gatcatcaagggccaagtgt_3′_ and PIM1Rw(mm) _5′_gatggttccggatttcttca_3′_ [53].

### Chromatin Immunoprecipitation

Chromatin immunoprecipitation was performed as described previously [54]. The DNA was immunoprecipitated with anti-HA antibody (Abcam, Cambridge, UK) and subsequently amplified with quantitative RT-PCR using SYBR Green QPCR Master Mix (Stratagene, La Jolla, CA) on a Stratagene-MX3005P under standard conditions. Results were quantified by SYBR Green Real-Time PCR analysis (each sample was performed in triplicate). The fold enrichment of immunoprecipitated samples was normalized on INPUT and expressed relative to the mock-treated control (IgG). Results were visualized after separating PCR products by agarose gel with etidium bromide staining. The primers used for the reactions were: ERG-ChIP-For _5′_GTGCTAGGCGAGTGGGAACAACTG_3′_ and ERG-ChIP-Rev _5′_AATGACCCAAATTCACCTCCTGAG_3′_ ; GAPDH_ChIP_For _5_′CCCAACTTTCCCGCCTCTC_3′_ and GAPDH_ChIP_Rev _5′_CAGCCGCCTGGTTCAACTG_3′_ .

### Western Blot analysis

Western Blot was performed as previously described [55] using the following antibodies: mouse anti-PIM1 (12H8) (Santa Cruz Biothecnology, CA, USA), mouse anti-cyclin B1 (H-433) (Santa Cruz Biotechnology, CA, USA), mouse anti-HA (Covance, Princeton, NJ, USA), rabbit anti-Actin (Sigma-Aldrich, St Louis, MO, USA).

### RNA interference

Short interfering RNA knockdown of PIM1 and ERG were performed with siRNA from the Thermo Scientific Dharmacon: siGENOME_SMART_ pool (003923-00) and siGENOME_siRNA (D-003886-01) for PIM1 and tERG, respectively. siGENOME_non targeting_siRNA (NT) was used as control (D-001210-01-05). siRNA were transfected in RWPE1 cells as indicated using Lipofectamine2000 (Invitrogen, Carlsbard, CA, USA). In PIM1 knockdown, cells were harvested after 72 hours from transfection for RNA isolation or whole cell lysates. For siERG experiments, transfections were performed as described in Tomlins et al. [18].

### Gene expression profile

RNA from each population of stable transfectants was extracted with Trizol®reagent (Invitrogen, Carlsbad, CA, USA) according to guidelines. Expression profiling was performed with Affymetrix-Genechip® Mouse Gene 1.0 ST Array according to manufacturer's protocol. *BRB-Array* tools was used to highlight differentially expressed genes. The array corresponding to one of the three tERG pools did not pass the quality control, and therefore was not included in subsequent statistical analysis. Note that ERG itself did not appear as an up-regulated gene in any dataset, as the ectopically expressed human ERG sequence does not hybridize to mouse ERG probe used in the array. Raw data have been deposited according to MIAME guidelines at NCBI Gene Expression Omnibus (GSE32481).

### Cell cycle and apoptosis analysis

For cell cycle analysis, cells were fixed in ethanol and stained with propidium-iodide (Sigma-Aldrich, St Louis, MO, USA). To evaluate apoptosis, Annexin-V assay was carried out with the Annexin V-FITC apoptosis detection kit (Bender MedSystems, Vienna, Austria) following the manufacturer's instructions. Flow-cytometry was performed on a Becton Dickinson FACSort by CELLQuest software (Becton Dickinson Immunocytometry Systems, Mountain view, CA, USA).

### Chemical compounds

Taxol and Idarubicin hydrochloride were obtained from Sigma-Aldrich and were dissolved at the desired concentration following guidelines.

### Thymidine uptake assay

Exponentially growing NIH-3T3 or RWPE-1 transfectants were plated in at least 4 replicates in 96 well plates at a density of 3000 cell/well. Where indicated, cells were treated with drugs or vehicle alone. 1 µCi of [^3^H]thymidine (Amersham) was added to each well 8 hours before harvesting onto glass fiber filters by a Tomtec automated cell harvester. Incorporation of [^3^H]thymidine was measured using a filter scintillation counter (1430 MicroBeta).

### Statistical analysis

GraphPad-Prism program was used to analyze the data. Two-tailed unpaired *t* test was used to determine statistical significance of the differences between data sets, where appropriate.

Spearman Rank correlation test was performed to analyze the correlation between ERG and PIM1 expression in the available prostate sample data-set (GSE14206) [34].
